# Teasing apart the impacts of curriculum and professional development on teaching assistants’ teaching practices

**DOI:** 10.1371/journal.pone.0262841

**Published:** 2022-02-09

**Authors:** Jenna Hicks, Jessica Dewey, Michael Abebe, Maxwell Kramer, Anita Schuchardt

**Affiliations:** 1 Office of Professional Development, University of Minnesota, Minneapolis, Minnesota, United States of America; 2 Biology Department, Syracuse University, Syracuse, NY, United States of America; 3 Department of Biology Teaching and Learning, University of Minnesota, Minneapolis, Minnesota, United States of America; University of Sydney, AUSTRALIA

## Abstract

Teaching assistants (TAs) often lead courses using curricula they did not design. Therefore, examining how curriculum and professional development (PD) interact to influence TAs’ teaching practices is critical. This study describes the effects of a curriculum and PD intervention in two contexts: when TAs are teaching curriculum that is explicitly linked to PD, and when teaching curriculum that is not linked to PD. The Intervention curriculum featured structured opportunities for reform-oriented teaching practices. The Intervention PD was situated in the context of these specific curriculum activities and modelled the desired teaching practices. TAs that participated in the intervention implemented more student-centered teaching practices than TAs that did not participate in the intervention, even when teaching curriculum that was not designed to be student-centered and was not linked to PD. A linear model of TAs’ teaching practices that included PD type, task cognitive demand and curriculum type indicates that cognitive demand has the largest relationship with teaching practices, followed by PD type. These results have implications for policy. They suggest that investment in curriculum-linked TA PD can be effective even when teaching curricula that is not linked to PD. Additionally, investment in development of higher-cognitive-demand tasks may be an effective strategy to support implementation of student-centered practices.

## Introduction

Undergraduate biology instruction is shifting from an instructor-centered to a student-centered pedagogical style that engages students in more cognitively demanding exploration of core concepts and practices in biology [1–4]. These curricular changes demand more of students and require elevated teaching practices [4, 5]. Teaching assistants (TAs) carry out much of the teaching associated with college laboratory courses [6], but knowledge of how to support TAs in implementing student-centered and cognitively demanding curriculum lags behind [10]. Few studies have examined the impacts of PD and curriculum on instructors’ teaching practices in higher education; a significant omission since the effectiveness of PD can depend on the curriculum [7, 8]. This report explores the effects of PD and curriculum on TAs’ teaching practices. We examine the effects of the PD program on TAs’ teaching practices in two contexts: 1) when teaching PD-associated versus un-associated curricular tasks, and 2) when teaching curriculum that varies in cognitive demand.

### Conceptual framework

**Fig 1:**
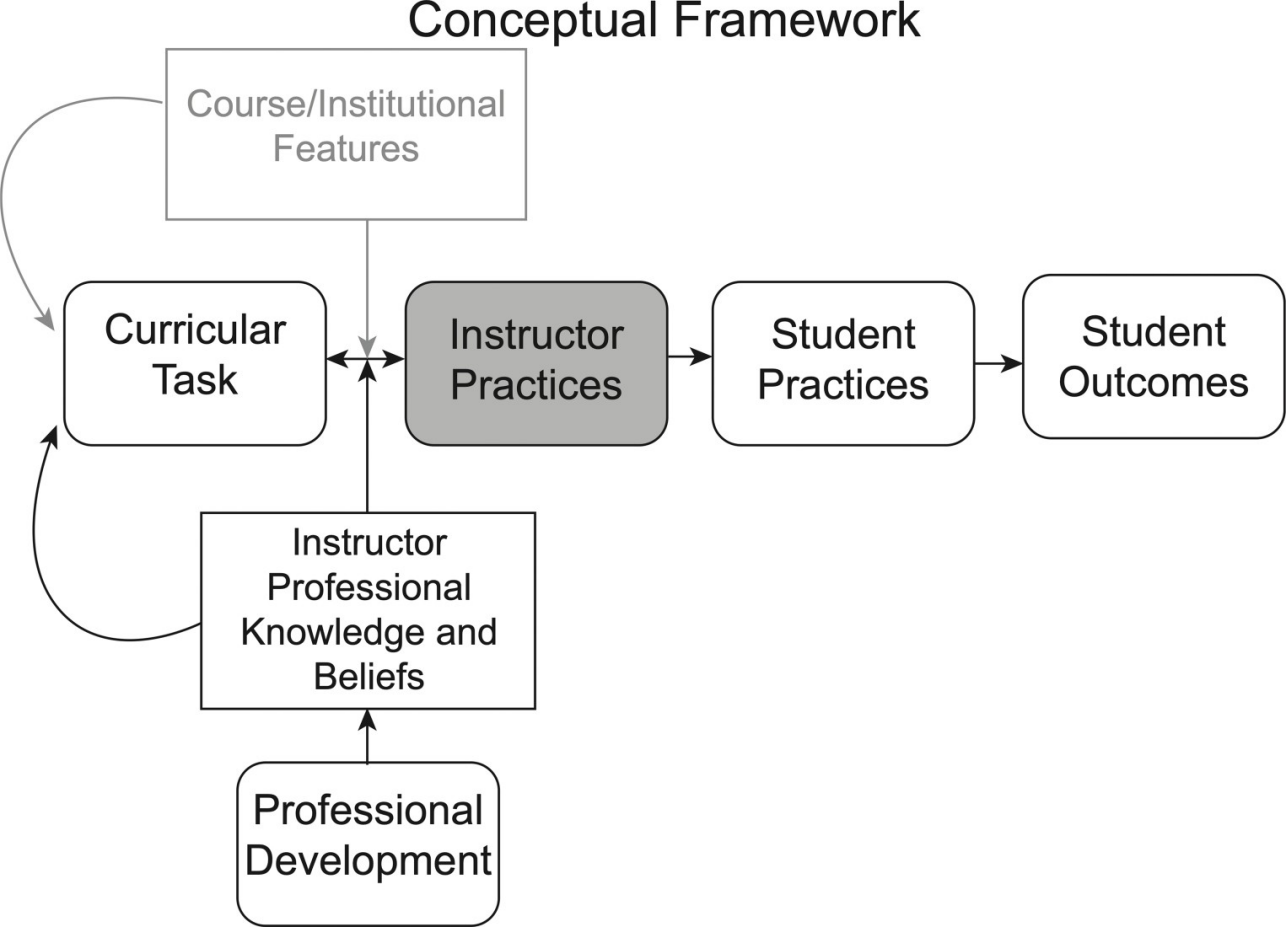
Conceptual framework. Adapted from Stein et al., [10] and Reeves et al., [12].

The conceptual framework guiding this study draws from work on professional development, teacher professional knowledge, and cognitive demand. Many factors converge to influence instructor teaching practices. Teaching practices influence students’ practices within the classroom, affecting student outcomes (Fig 1) [9–11]. In this study, institutional and course context does not vary, so this box has been greyed out in Fig 1. Instructors’ teaching practices are critical in directing students’ interactions and cognition in the classroom, and are the focal point of this framework [10].

Instructors’ teaching practices consist of the actions implemented to facilitate instruction, including lesson planning, in-person instruction, and assessment [12]. Instructors’ practices can be influenced by instructors’ professional knowledge, attitudes, and beliefs, as well as the curricular tasks. Curricular tasks provide the initial plan for the content, structure, and cognitive demand of the lesson [13] (Fig 1). The instructor’s professional knowledge consists of their understanding of pedagogical techniques, students’ ideas about the subject matter, and the concepts within the task [12, 14, 15]. Instructors’ professional knowledge, attitudes, and beliefs may influence how they plan and implement curriculum [12]. PD is designed to shift elements of instructors’ professional knowledge, attitudes, and beliefs to affect instructors’ teaching practices (Fig 1). However, changes in instructors’ knowledge, attitudes, and beliefs may not always lead to changes in teaching practices [7, 14, 16]. This study investigates the impact of PD and curriculum on TAs’ teaching practices following an instructional intervention that manipulates features of the curriculum and PD.

### Literature review

PD has been well-studied in the context of K-12 teachers, and elements of effective PD for K-12 teachers have been identified, including: PD should be linked to classroom lessons and focus on subject matter content, PD should model teaching strategies that teachers are asked to use with their students, and PD should engage teachers in reflection [17–22]. The elements of effective PD identified for K-12 teachers are effective despite myriad school contexts, suggesting that these elements may also be effective in structuring TA PD [18, 20, 21].

In contrast to the large body of literature on K-12 teacher PD, fewer studies have investigated PD for TAs [12]. Most of these studies have examined changes in TAs’ attitudes and beliefs, or their self-perceptions of their knowledge and skills. These studies have found that PD can increase TAs’ teaching self-efficacy, knowledge of pedagogical strategies, and beliefs about how people learn [23–32]. Few studies have examined the effects of PD on TAs’ instructional practices [33–36], which is critical because changes in beliefs and knowledge are not always translated into changes in practices [7, 14, 16].

Many graduate students report that they do receive PD for teaching, but state that these PD programs do not provide adequate support for developing pedagogical skills [26, 37]. One-time, pre-semester workshops are the predominant mode of PD for TAs [37], despite studies suggesting that one-time workshops are often ineffective in changing instructors’ teaching practices [38]. Sustained, multi-session PD programs can be successful in shifting TAs’ ideas about teaching, and/or TAs’ teaching practices in the classroom [24, 25, 29, 35, 36, 39–41]. For example, multi-session PD programs have resulted in increased use of student-centered teaching practices [36], changes in TAs’ attitudes and ideas about teaching [29], and TAs reporting increased knowledge of topics covered during PD [39]. Many of these programs run for a full semester, requiring double-digit hours of contact time between TA participants and PD facilitators. While effective, this lengthy time commitment may pose a barrier for implementation of similar programs at many institutions [42]. It is necessary to identify effective PD strategies that can be implemented within time constraints that are a reality for participants and facilitators.

Multiple reports of sustained, multi-session PD for TAs describe programs that are directly linked to the curriculum for a specific course [31, 34–36, 43]. Most of these programs exceeded 20 hours (average 37 hours). Three studies investigated the effect of course-linked PD on TAs’ teaching practices, and all found evidence that TAs incorporated student-centered practices learned during PD into their teaching [34–36]. These studies did not examine whether TAs implemented these student-centered teaching practices in lessons that were not related to PD [34–36]. Situating PD within the instructors’ teaching context is also an evidence-supported strategy for structuring effective PD for K-12 teachers [18, 44]. However, few studies across the K-16 spaces have investigated to what extent curriculum-linked PD effectively prepares instructor participants to transfer newly developed ideas and practices to teaching curriculum that was not linked to PD.

Student-centered curricula push students to engage with core concepts and practices of a discipline through scientific inquiry [1, 2, 45]. This style of instruction asks students to construct explanations of phenomena and use evidence to support claims, increasing the cognitive demand on students [10, 13, 45, 46]. Curricular tasks exist on a spectrum of cognitive demand. To compare cognitive demand across curricular tasks, the Task Analysis Guide in Science (TAGS) framework breaks this spectrum of cognitive demand into five levels [45].

An example of a high-cognitive-demand task (level 5 of 5) is one where students tackle an open scientific question with very little prescribed structure or directives from the instructor (described as *Doing Science* by Tekkumru-Kisa et al. [45]). This type of task requires students to use discipline-relevant practices to deepen their understanding of a scientific phenomenon [10, 45, 47, 48]. An example of a low-cognitive-demand (level 1 of 5) task is a memorization activity, where students are required to remember previously introduced definitions of scientific content or practices. Memorization does not require students to have a deep understanding of the content or practices, and can provide opportunities for students to practice new skills or remember basic facts [10, 45]. However, low-cognitive-demand tasks are less effective in helping students generate and retain knowledge [49]. These examples represent the highest and lowest ends of the cognitive demand spectrum, respectively. Additional gradations of cognitive demand exist between these endpoints, with less prescriptive structure and more space for inquiry occurring as tasks progress from lower- to higher-cognitive demand (Table 1) [45].

**Table 1:** Descriptions and examples of TAGS levels.

TAGS Level	Description	Example
**5** Doing Science Tasks	An open-ended task that asks students to use scientific practices to deepen their understanding of content with little guidance from the instructor.	Designing and carrying out an experiment to test a student-generated question about a scientific phenomenon.
**4** Tasks Involving Guidance for Understanding–Guided Integration	Tasks with a suggested pathway that require cognitive effort on the part of the students to use a scientific practice to understand content.	Designing elements of an experiment (e.g., controls, dosage amounts) within a provided experimental methodology to test an instructor-generated question about a scientific phenomenon.
**3** Tasks Involving Guidance for Understanding–Guided Practices and Content	Tasks with a suggested pathway that require cognitive effort on the part of the students to engage in developing understanding of a scientific practice **or** scientific content.	Applying understanding of the mathematical structure of the t-test to determine whether two samples are likely to be significantly different based on graphs of mean and variation.
**2** Tasks Involving Scripts	Students follow a script to work on practices, content or practices related to content.	Using provided computer code to calculate the results of a statistical test.
**1** Memorization Tasks	Students are asked to memorize content or practices.	Labeling a diagram of a cell in preparation for a quiz.

Examples for Levels 2–4 are derived from the Traditional and Intervention curriculum. All descriptions and the examples for Levels 1 and 5 that did not occur in the planned curriculum in this study came from the manuscript by Tekkumru-Kisa and colleagues [45].

There may be an association between cognitive demand on students and teaching practices [11, 50]. Cognitively demanding tasks can result in frustration while students grapple with unfamiliar concepts or contexts. Some instructors respond to this frustration by taking over challenging elements of tasks, thereby reducing cognitive demand and subsequent opportunities for student learning [11]. Implementing and maintaining student-centered pedagogical practices is associated with maintaining a high level of cognitive demand [10, 11]. Instructors’ teaching practices have a clear impact on the enacted cognitive demand of a task [10, 46, 51]. However, the relationship between planned cognitive demand (as written in curricular materials) and instructors’ enacted teaching practices is underexplored.

### Study context and objective

In this study, we implement a multi-session curriculum-linked PD program that is relatively short in duration (4 hours). Elements shown to be effective for K-12 teachers and TAs (modelling of teaching practices, structured opportunities for TAs to reflect on the modelled teaching practices, and a direct link to classroom content) were included in this PD intervention [18, 21, 31, 36]. This PD intervention is explicitly linked to a companion curriculum intervention consisting of five short (30-minute) tasks that supplement regular laboratory exercises in an undergraduate introductory biology laboratory course [52]. These tasks are designed to guide student inquiry and promote TAs’ use of student-centered teaching practices to facilitate the activities. Part of this curriculum focuses on statistics, a difficult topic for both students and experts [53, 54], further necessitating PD that incorporates both the implementation of teaching strategies and the curriculum subject matter [55, 56]. We first investigate the effectiveness of this reduced-length, multi-session Intervention PD on TAs’ teaching practices in two instructional contexts: when teaching PD-linked Intervention curriculum, and when teaching Traditional curriculum that was not linked to PD. We then investigate the relative contributions of the PD intervention and cognitive demand of curricular tasks (which varies within the Intervention and Traditional curriculum) on TAs’ teaching practices. Specifically, this study aims to answer these research questions:

1. To what extent do the intervention curricular activities and PD prepare TAs to implement student-centered teaching practices in the classroom?
2. To what extent do TAs that participated in the curriculum/PD intervention implement student-centered teaching practices when teaching curriculum that was not linked to PD?
3. To what extent do PD and the cognitive demand of the curricular tasks impact teaching practices of TAs when controlling for curriculum type?

## Methods

### Study design

This study was conducted within a larger study on the impact of a curriculum intervention within an undergraduate introductory biology laboratory course across three semesters at a large, American, Ph.D.-granting university. This study was approved by the University of Minnesota Institutional Review Board ID#: STUDY00003137. Consent followed the process approved by the IRB. TAs were provided with information about the study on the BioVEDA survey and an introductory email. They were asked on the BioVEDA survey whether they agreed to have their observation and survey data included in the study. TAs were also informed that they could opt out of the study at any time by contacting one of the researchers.

**Fig 2:**
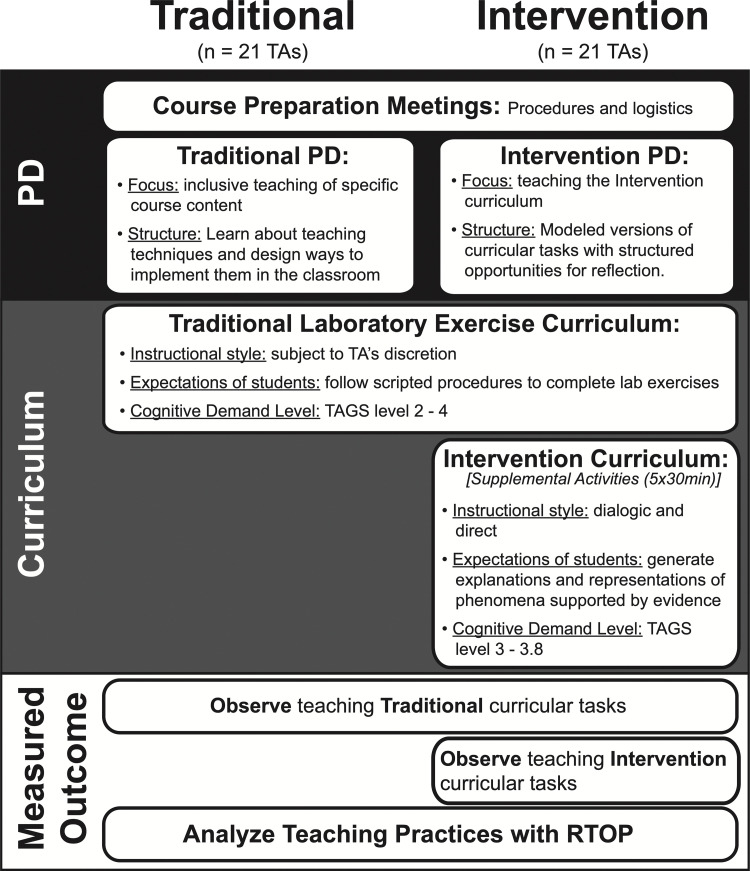
Study design. All TAs participated in course preparation meetings and taught the traditional laboratory exercise curriculum. TAs participated in Traditional or Intervention supplemental PD. Intervention TAs taught supplemental Intervention curriculum activities. Cognitive demand level is measured by the Task Analysis Guide in Science (TAGS; see the ‘Cognitive Demand’ section for detail). All participants were observed, and audio recorded while teaching. Recordings were subsequently analyzed using the Reformed Teaching Observation Protocol (RTOP; see the ‘Teaching Practices’ section for detail).

The course was divided into laboratory sections each containing approximately 20 students. Half the sections were randomly designated as ‘Traditional’ instruction sections, and the remaining sections were designated as ‘Intervention’ sections. TAs in Intervention sections (N = 21; hereafter, ‘Intervention TAs’) implemented five short (~30 minute) Intervention curricular tasks in addition to the typical laboratory exercises. TAs in Traditional sections (N = 21; hereafter, ‘Traditional TAs’) only implemented the typical laboratory exercises. All TAs participated in course preparation meetings designed to support procedural implementation of the typical laboratory exercises. Intervention TAs also participated in four additional hours of PD that was designed to support implementation of Intervention tasks, while Traditional TAs participated in time-matched PD that focused on inclusive teaching (Intervention and Traditional curriculum and PD are described below). Fig 2 presents an overview of the study.

This study was a collaboration between the course coordinators and the research team. The research team designed the Intervention curriculum and PD. The course coordinators designed the Traditional curriculum and PD. The research team had no day-to-day involvement in hiring TAs, the Traditional course curriculum or facilitation of laboratory preparation meetings.

### Participants

Study participants included one teaching specialist and 41 graduate students who were paid TAs for an introductory biology laboratory course. TAs were randomly assigned to Traditional or Intervention sections by a course coordinator who was not directly involved in this study. There were no statistically significant differences between Traditional and Intervention TAs in the number of years in graduate school, TA experience, or gender (Table 2). Intervention TAs scored higher than Traditional TAs on a pre-semester content knowledge assessment (BioVEDA, described below).

**Table 2:** Participant demographic information.

Demographic Characteristic	Traditional	Intervention	Statistic	*p*-value	Effect Size
**Years of graduate School**	3.6±1.8	3.2±1.7	*W* = 201	*p* = 0.63	*d* = 0.2
**Semesters of TA experience**	4±2.2	3±2.0	*W* = 167	*p* = 0.17	*d* = 0.5
**Number of female TAs**	*N* = 15/21	*N* = 11/21	χ^2^(1) = 0.9	*p* = 0.34	*V* = 0.15
**BioVEDA assessment score**	64±15%	75±15%	*W* = 140	*p* = 0.04	*d* = 0.7

Values are presented as Mean±SD, unless otherwise indicated. *W* statistics were calculated via a Wilcoxon ranked-sum test, and χ^2^ statistics were calculated via a Chi-square test. Effect sizes are denoted as *d* (Cohen’s *d*) or *V* (Cramer’s *V*).

### Curriculum

The Traditional core curriculum consisted of largely scripted laboratory exercises based on a lab manual detailing procedures that students completed in class (hereafter, the ‘Traditional curriculum’). Content covered by the Traditional curriculum included laboratory techniques, experimental design, and data analysis. All TAs (Traditional and Intervention) taught the Traditional curriculum. Instructional style was not prescribed for TAs.

The Intervention curriculum consisted of five short tasks that supplemented the Traditional curriculum during the first third of the semester in Intervention laboratory sections. The supplemental Intervention tasks comprised approximately 3% of the total instructional contact hours across the semester, and the remaining 97% of instructional time was spent on Traditional tasks. The content covered by the Intervention curriculum complemented the laboratory exercises specified in the Traditional curriculum. A detailed explanation of the Intervention curriculum is published elsewhere [52]. Table 3 summarizes features of the Traditional and Intervention curricula.

**Table 3:** Features of traditional and intervention curricula.

Curriculum Feature	Traditional Curriculum *(Taught by all TAs)*	Intervention Curriculum *(Taught by Intervention TAs)*
Instructional style	Direct	Dialogic and direct
TAs’ role	Explain, demonstrate, guide	Facilitate student production of ideas, guide, explain
Expectations of students	Follow explicit procedures to complete laboratory exercises	Generate explanations and representations of phenomena supported by evidence
Instructor supports	Procedural (written procedural guide, PowerPoint slides)	Educative (Curriculum design rationale, scientific and statistical concepts, student thinking, rationale for and tips on how to implement teaching techniques)

Table adapted from Remillard et al. [13].

For Intervention curricular tasks, Intervention TAs were provided with instructor supports modelled after educative curricular materials (ECM) used for K-12 teachers [57]. The ECM consisted of an overview detailing the learning objectives and design rationale for the task; lesson plans describing how to implement each task with callout boxes highlighting information about pedagogical techniques, student thinking, or specific learning goals; and copies of student worksheets (see example ECM in the S1 File).

### Professional development

All TAs attended weekly preparation meetings (~25 hours per semester) where course coordinators lectured on logistics, procedures, and content. All TAs also participated in 4 hours of Traditional or Intervention PD that occurred over 4 sessions during the first third of the semester.

Traditional and Intervention PD differed in topic and structure. Traditional PD focused on shifting the attitudes and interest of TAs and their students about course content to make laboratory exercises more inclusive and accessible. Targeted course content varied by semester and was chosen by the course coordinator. In these sessions, TAs learned about teaching techniques and then designed ways to implement them in the classroom.

Intervention PD focused on preparing TAs to teach the Intervention curricular tasks and aimed to elevate TAs’ knowledge of subject matter within the tasks, and knowledge of teaching practices that should be used during task enactment. Facilitators presented each task’s learning goals. Trainings were structured as modelled versions of curricular tasks, with facilitators acting as TAs, and TAs acting as students. Facilitators modelled teaching strategies that TAs could use to enact the tasks. TAs were asked to reflect on their thought process as they participated in the tasks, note any difficulties they had, anticipate areas of difficulty for students, and reflect on teaching practices that facilitators implemented.

The inclusion of modelled teaching practices linked to specific tasks and opportunities for reflection are evidence-supported practices in K-12 PD and represent key differences between Intervention and Traditional PD [18, 21]. While both groups received instruction on teaching practices, Traditional TAs did not experience these practices as students or see an example of how one might implement these practices.

Intervention PD facilitators were members of the research team and part of the curriculum development team. These facilitators consisted of a Ph.D. student in a STEM Education program and a postdoctoral associate with a biology Ph.D. and one year of biology education research experience. Traditional PD facilitators consisted of a course coordinator (teaching faculty) and a member of the research team (education research faculty, who was also involved in development of the Intervention curriculum).

### Data collection

To examine how PD and curriculum impacted classroom practices, TAs were audio recorded teaching Traditional and Intervention curricular tasks (a maximum of two times per semester). TAs were assured that the research team would not share any identifiable information with persons involved in managing or hiring for TA appointments. Traditional TAs were recorded teaching Traditional tasks and Intervention TAs were recorded teaching both a Traditional and an Intervention task.

### Measures

The Reformed Teaching Observation Protocol (RTOP) was used to measure teaching practices that TAs enacted in the classroom [58]. The RTOP was chosen because it accounts for instructor and student behaviors in a detailed way that goes beyond broad categorization of actions. The RTOP has been extensively validated and used to analyze the degree of student-centered teaching used by K-12 teachers, university faculty, and teaching assistants [14, 36, 58, 59]. The RTOP is a holistic measurement tool containing 25 items that are rated on a 0-4-point scale: 0 indicates the item never occurred, and 4 indicates the item was very characteristic of the lesson. Total RTOP scores (summed across all 25 items) were used to measure TAs’ instructional practices.

A research assistant who was not involved in the design or implementation of the curriculum or PD was the primary coder of the audio recordings of classroom lessons. A second coder independently rated 45% of the audio recordings of classroom lessons. The second coder was involved in the design and implementation of the curriculum and PD. Both coders were blinded to the identity of the TAs, their Traditional/Intervention group status, and Traditional/Intervention designation of the recorded curricular task. The intra-class correlation coefficient for the two coders is 0.75 (95% CI = [0.68, 0.81]), indicating excellent inter-rater agreement [60].

This study assessed the cognitive demand of the tasks that were taught by TAs during the recorded lessons. Cognitive demand was determined by using the Task Analysis Guide in Science (TAGS) framework [45] to analyze the curricular materials for that task (e.g. lesson plans, student laboratory manuals and/or worksheets). Tasks were subdivided into activities which were categorized using TAGS by two independent coders (67% agreement). An overall cognitive demand score for each task was calculated by averaging the cognitive demand levels across individual activities within the task and categorizations were averaged across two independent coders. On a five-point scale, the planned cognitive demand of the tasks in the curricular materials ranged from an average of 2 (scripted tasks) to 4 (guided tasks that integrate scientific content and scientific practices). Table 1 provides an example of a task at each of these TAGS levels. Most tasks were either a level 2 or 3.

The Biological Variation in Experimental Design and Analysis (BioVEDA) assessment was used to measure TA content knowledge because it can be used to evaluate respondents’ understanding of variation in experimental design and data analysis, which is a learning goal for this course [61]. All TAs completed the BioVEDA assessment before the start of the semester.

### Analyses

Differences in RTOP scores across PD and curriculum type were examined via one-way ANOVA. Assumptions of ANOVA were met (independence and normality of samples, homogeneity of variance). Post-hoc analyses were conducted via Tukey’s HSD. Effect sizes have been provided for comparisons between groups (Cohen’s *d*).

To investigate the relative impact of the supplemental PD (Traditional or Intervention) and cognitive demand of the task (measured by TAGS) on classroom teaching practices (measured by RTOP) while controlling for curriculum type (Traditional or Intervention), linear models were fitted to the data using forward and backward stepwise regression. Assumptions of linear regression (linear relationship of residuals, normally distributed residuals, homoscedasticity of residuals, absence of highly influential cases) were considered satisfied after graphical examination. The assumption of multicollinearity was met (Variance Inflation Factor (VIF) is 3.6) [62].

An alpha level of .05 was used to determine statistical significance for all analyses. Data used in these analyses has been provided in a CSV file in (S1 File).

## Results

**Fig 3:**
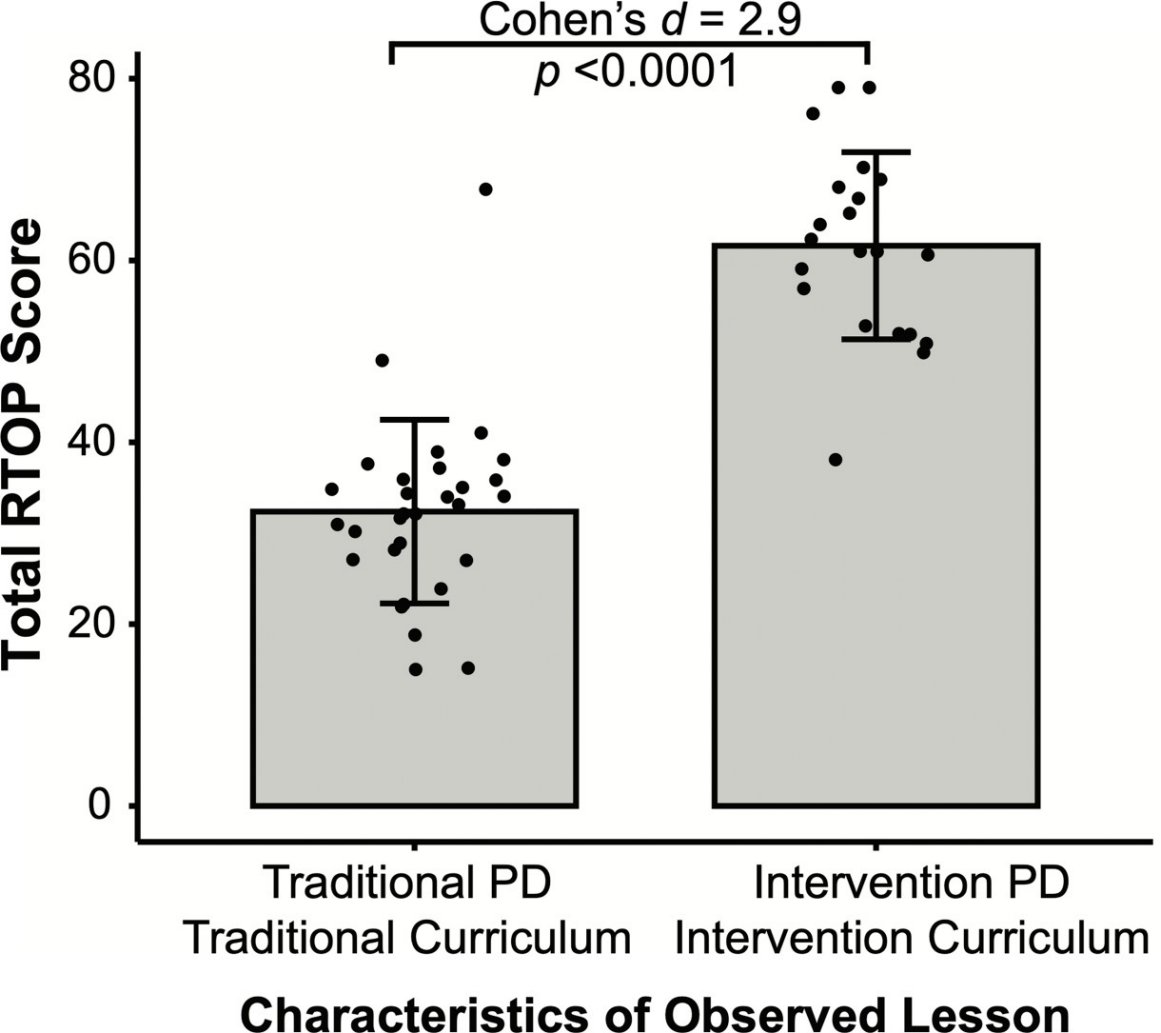
Intervention PD and curriculum elicit more student-centered teaching practices from TAs. Total RTOP score from TAs teaching Traditional tasks that participated in Traditional PD (left; *N =* 30), and TAs teaching Intervention tasks that participated in Intervention PD (right, *N* = 21). Each dot indicates one observation of one TA. Bar±error bars = mean±standard deviation.

To investigate the effect of Intervention curriculum and PD on TAs teaching practices, total RTOP scores were compared between Traditional TAs teaching Traditional tasks and Intervention TAs teaching Intervention tasks. The difference in total RTOP scores between these groups is significant (*F*(1, 49) = 101.9, *p* < .001, Cohen’s *d* = 2.9; Fig 3). The mean RTOP score for Traditional TAs teaching Traditional tasks (*M* = 32.4) represents lecture-style instruction with minor student participation [16] (Fig 3). The mean RTOP score for Intervention TAs teaching Intervention tasks (*M* = 61.6) represents active student participation in critiquing or carrying out investigations [16] (Fig 3).

**Fig 4:**
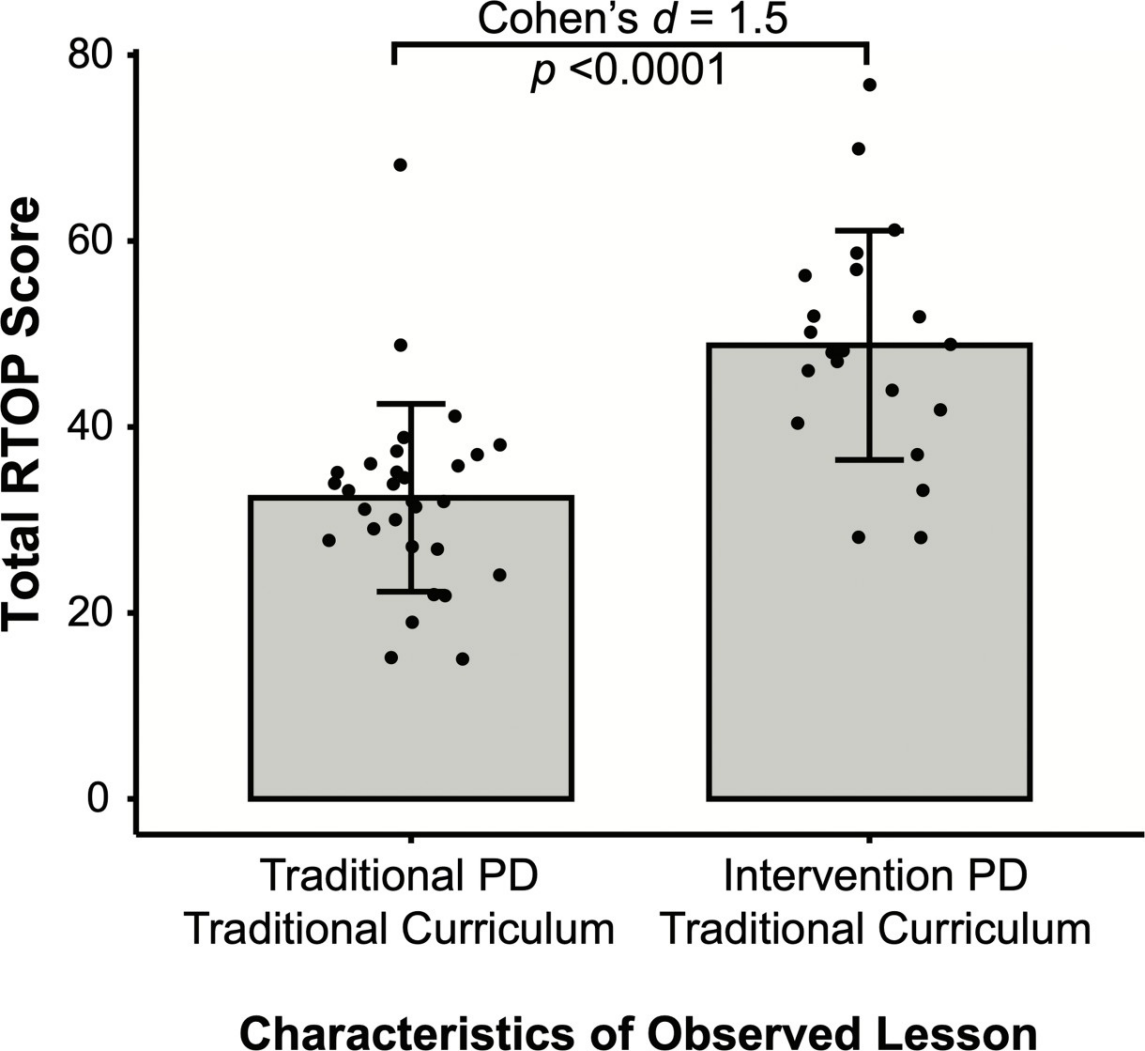
Intervention TAs transfer student-centered teaching practices to traditional tasks. Total RTOP score from TAs that participated in Traditional PD (left; *N =* 30), and TAs that participated in Intervention PD (right, *N* = 21). All observations shown represent Traditional curricular tasks. Each dot indicates one observation of one TA. Bar±error bars = mean±standard deviation.

Intervention PD was focused solely on preparing TAs to teach Intervention curricular tasks. Curriculum developers and PD facilitators neither asked nor specifically encouraged Intervention TAs to continue to implement student-centered teaching practices when teaching Traditional tasks. Interestingly, Intervention TAs still have 1.5-fold higher RTOP scores than Traditional TAs, even when teaching Traditional tasks (*F*(1, 49) = 27.1, *p* < .001, Cohen’s *d* = 1.5; Fig 4). When teaching Traditional tasks, the mean RTOP score for Traditional TAs (*M* = 32.4) represents lecture-style instruction with minor student participation, whereas the mean RTOP score for Intervention TAs (*M* = 48.8) represents ‘significant student engagement with some minds-on as well as hands-on involvement’ [16] (Fig 4).

Planned cognitive demand (as written in the curricular materials) varied within both the Intervention and Traditional curricula. Traditional and Intervention curricular tasks differed in multiple ways, including instructional style, expected role of the TA, expectations of students, and the type of instructor supports provided (Table 3). Therefore, task curriculum type was used as a control variable when examining the relative contributions to teaching practices of planned cognitive demand, and PD type (Traditional versus Intervention) using linear regression models. TAs’ content knowledge (measured by pre-semester BioVEDA assessment score) was also included as a control variable, since Intervention TAs had significantly higher BioVEDA scores than Traditional TAs.

In the best fitting model, cognitive demand, curriculum type, and PD type (*F*(3, 68) = 59, *p* < .001, *R*^*2*^ = .72), accounted for approximately 70% of the variation in RTOP scores. Content knowledge (BioVEDA score) was not a significant contributor to teaching practices. When controlling for curriculum type, planned cognitive demand of the task (*ß* = .53, *η*^2^ = .35) had a two-fold larger contribution to TA teaching practices than PD type (*ß* = .29, *η*^2^ = .15) (Table 4).

**Table 4:** Regression coefficients and statistics for predictor variables of total RTOP score.

Predictor Variable	*B*	SE (*B*)	*ß*	*η* ^2^	*p*-value
Task Cognitive Demand	13.35	2.18	.53	.35	< .0001
PD Type (Traditional or Intervention)	9.47	2.73	.29	.15	< .0001
Task Curriculum Type (Traditional or Intervention)	5.78	2.94	.16	.05	.054

*R*^*2*^ = .72, *p* < .0001.

Abbreviations: *B* = unstandardized coefficient, *ß =* standardized coefficient, *η*^2^ = partial eta-squared.

## Discussion

Multiple factors can affect instructors’ teaching practices [36, 46, 63]. This study examined the effect on TAs’ teaching practices of different types of PD and the planned cognitive demand of student activities. Across all observations, TAs that participated in Intervention PD used more student-centered teaching practices than TAs that participated in Traditional PD. In Intervention PD, facilitators modeled student-centered teaching practices while TAs engaged in Intervention curricular tasks as students. Facilitators led discussions where TAs reflected on the benefits of the teaching strategies used by the facilitators and how they could implement them in their classes. During Traditional PD, TAs learned about inclusive teaching strategies and designed plans for using them in their classes; but the facilitators did not model these strategies for the TAs. Modeling of student-centered teaching practices and providing opportunities for reflection are strategies that have been shown to be effective for changing teaching with K-12 teachers [15–20] but have not been used in studies of the effect of PD on TA teaching practice [10, 32–34]. Investigations into the impact of PD on TA teaching practice have generally included PD that includes more contact hours than the PD in this study [32–34]. The results from this study suggest that modeling of teaching strategies combined with reflection can lead to changes in TA teaching practices with as few as six contact hours. However, as discussed below, our results suggest that alignment of the curriculum and PD and the cognitive demand of the curriculum are also factors affecting TA teaching practice.

Intervention TAs had the highest RTOP scores when they were teaching Intervention curriculum. The RTOP scores were lower when Intervention TAs were teaching Traditional curriculum. These findings are consistent with those of Addy & Blanchard [7]. They found reform-oriented PD for TAs was effective in shifting TAs’ beliefs about teaching and learning, but the structure of some laboratory tasks constrained TAs’ enactment of student-centered teaching practices, resulting in implementation of more instructor-centered practices [7]. In this study, Intervention PD was aligned with the Intervention curriculum, communicating similar objectives, and prioritizing generation of ideas by students combined with consensus-building through small group and large group discussion. Therefore, Intervention curriculum and PD could work together to support TAs in implementing student-centered teaching practices. Together, these studies suggest that student-centered teaching practices are more likely to be observed when TA PD and the structure of the curricular tasks align on this dimension. Therefore, investing resources to provide reform-oriented PD for TAs may be inefficient if curricula constrain implementation of student-centered teaching practices.

Significantly, Intervention TAs still used more student-centered teaching practices than Traditional TAs on Traditional tasks not covered in either PD. Intervention TAs were not asked to implement student-centered teaching practices when teaching the observed Traditional tasks. This result suggests that Intervention TAs are independently transferring their knowledge about teaching learned during the Intervention PD to a novel curricular task.

TAs’ knowledge about the subject matter covered by the curriculum was not a direct predictor of RTOP score. Other studies have shown that increased subject matter knowledge does not necessarily correspond to more effective instruction [64, 65]. The lack of a direct association between content and RTOP score may be because PCK, TAs understanding of how to make the content accessible to students, was not measured [10, 12, 13]. While an instrument to measure PCK of statistics for middle school teachers has been developed [66], there is not yet a publicly developed instrument for measuring PCK of statistics used in biology laboratory settings. In this study, both the Intervention curriculum and PD contained features intended to support TAs who were less familiar with the content as well as the pedagogy within the Intervention curriculum. Intervention TAs received ECM which have been shown to be effective in elevating instructors’ content knowledge and PCK [57, 67, 68]. During Intervention PD, TAs experienced Intervention curricular tasks as students, which may have increased their understanding of PCK as well as the content.

The effect of instructors’ teaching practices on the cognitive demand of an activity is well-studied [10, 11, 69]. This study investigates the reciprocal relationship: how the planned cognitive demand of a task contributes to instructors’ teaching practices. Planned cognitive demand varied across both Traditional and Intervention curricular tasks in this study. In regression models that examined relative contributions of cognitive demand and PD type, while controlling for curriculum type, cognitive demand of the task had the strongest relationship to TAs’ teaching practices. These results suggest that tasks with higher cognitive demand could prompt TAs to implement student-centered teaching practices.

The strong relationship between the planned cognitive demand of a task (measured by TAGS) and TAs’ teaching practices (measured by the RTOP) is not simply a reflection of alignment between these instruments. Both the TAGS framework and the RTOP instrument award higher measures to lessons that are student-directed and inquiry-oriented [47, 58]. However, planned high cognitive demand is not always a predictor of student-centered teaching [51]. Moreover, these instruments are not entirely convergent. For example, the RTOP also privileges (among other lesson features) the use of multiple representations and multiple modes of communication [58]. Neither of these features are referenced by TAGS, suggesting that the strong relationship between the planned cognitive demand of the lesson and TAs’ teaching practices is not merely a reflection of alignment between instruments. This strong relationship is also not due to a confound between curriculum type and cognitive demand because curriculum type was controlled for in the regression analyses and cognitive demand varies within both curriculum types.

The observed tasks comprised only part of the range of the levels of cognitive demand developed by Tekkumru-Kisa et al. [45]. None of the recorded tasks were on the lowest end of the cognitive demand spectrum (memorization tasks, Level 1), or the highest end (doing science, Level 5). The relationship between cognitive demand and teaching practices may be weaker for very cognitively demanding tasks because novice instructors may not have the pedagogical toolkit required to facilitate these tasks [51]. Future studies should investigate the relationship between planned cognitive demand of the curricular task and TAs’ enacted teaching practices across all levels of cognitive demand.

### Implications

These results suggest ways to prioritize resource investment when designing TA PD. While pre-semester orientations are a popular, low resource-investment form of PD [37], studies indicate that one-time workshops are less effective than PD that takes place in multiple sessions over a longer time period [18, 38]. Multi-session, curriculum-linked PD programs have been effective in shifting TAs’ teaching practices for the curriculum covered during PD [34–36]. However, multi-session, curriculum-linked PD in published studies typically requires more time investment and is tailored to a specific group of instructors [31, 34–36] Thus, there are concerns about investing resources in preparing TAs for a narrow range of teaching experiences. Curriculum-linked PD may be necessary though when the content is unfamiliar to instructors [55, 56]. The Intervention PD described here focused exclusively on teaching Intervention curricular tasks, yet Intervention TAs still showed gains in teaching practices when teaching Traditional curricular tasks compared to Traditional TAs. These results show that curriculum-linked PD can be short and still be effective. Moreover, TAs can transfer teaching practices learned in curriculum-linked PD to teach other curricular tasks that were not covered during PD suggesting that curriculum-linked PD can be an effective investment of resources.

Student-centered teaching practices facilitate the implementation of a higher-cognitive demand curriculum [11] and this study shows that the cognitive demand of the curriculum is associated with greater use of student-centered teaching practices. Combined, these studies suggest that to be most effective, PD should be accompanied by redesign of materials so that they are cognitively demanding. This may be one way to support both instructors and TAs in changing to a more student-centered pedagogy, which has been associated with improved student performance [70].

### Study limitations and future directions

This study presents evidence indicating that Intervention PD was a significant predictor of TAs’ implementation of more student-centered teaching practices. However, several features of the Intervention PD differed from features of Traditional PD. Therefore, teasing apart the effects of individual features of the Intervention PD awaits further research. Moreover, the study reported here was for one curriculum context (statistics in a laboratory setting) at one institution. Additional studies are needed to see if the findings generalize across contexts and institutions.

Individual TAs have differing experiences, motivations, and abilities regarding teaching [10]. Any of these factors could have affected their teaching. This study showed that content knowledge did not have an impact on TA teaching practices, in agreement with other studies [55–62]. Future studies are needed to explore the impact of the other factors (including PCK) on TAs’ teaching practices after PD. Additionally, individual TAs may experience different motivations and/or comfort with the content on different days and this could impact their teaching. One limitation of this study is that the claims are based on one to two samples of each individual’s teaching. However, claims are based on observations of many TAs mitigating against individual variation. Examining the extent of variation in individual TAs teaching and the factors responsible for that variation is an important avenue for future research.

This study suggests that TAs are transferring knowledge learned during Intervention PD to activities that are not related to the PD sessions. This inference is made based on observations of teaching analyzed using the RTOP, which limits the ability to draw stronger conclusions about how TAs may be applying knowledge about teaching gained from PD to novel activity contexts. Future studies should include qualitative analysis of interviews with TA participants to gain greater insight into TAs’ choices about whether and how to apply student-centered teaching practices to activities that were not part of PD.

## Conclusions

The demand for student-centered, inquiry-driven science instruction is increasing. This kind of instruction is cognitively demanding for students and requires instructors to implement student-centered pedagogical practices. This study indicates that reform-oriented curriculum implemented alongside curriculum-linked PD supports TAs’ enactment of student-centered teaching practices, with a relatively short time investment. The structure of PD appears important: TAs that participated in curriculum-linked PD that incorporated modelling of teaching practices and instructor-driven reflection about those practices (Intervention PD) taught curricular tasks in a more student-centered manner than TAs that participated in PD in which instructors learned about teaching practices and designed small changes to the traditional curriculum to incorporate those strategies (Traditional PD). Curriculum-linked (Intervention) PD was not only effective in supporting TAs’ in using more reform-oriented teaching practices for the tasks covered during PD; TAs also transferred student-centered teaching practices learned during PD to novel lesson contexts. This study suggests that curricular tasks that are planned to be cognitively demanding are associated with implementation of student-centered teaching practices. These data indicate that student-centered teaching practices can be supported by PD situated in content that instructors will be teaching and by cognitively demanding curricula.

## Supporting information

S1 File TA data. This csv file contains information on each TA’s BioVEDA score, RTOP score for each observation. The file also contains the average cognitive demand TAGS level for each observed activity.
(CSV)
